# Risk factors for rehospitalization after trauma: a follow-up study

**DOI:** 10.1007/s00068-025-02944-1

**Published:** 2025-08-11

**Authors:** Bella Savitsky, Arielle Kaim, Irina Radomislensky, Arnona Ziv

**Affiliations:** 1 Israel National Center for Trauma & Emergency Medicine Research, The Gertner Institute for Epidemiology and Health Policy Research, Sheba Medical Center, 52621 Tel-Hashomer, Israel; 2 Department of Emergency and Disaster Management, School of Public Health, Gray Faculty of Medical and Health Sciences, Tel Aviv University, Tel Aviv, Israel; 3 School of Health Sciences, Ashkelon Academic College, Yitshak Ben Zvi 12, Ashkelon, Israel; 4 Data & Analytics Division, The Gertner Institute for Epidemiology and Health Policy Research, Sheba Medical Center, 52621 Tel-Hashomer, Israel

**Keywords:** Rehospitalization, Trauma, Injury, Trauma registry

## Abstract

**Introduction:**

Unplanned rehospitalizations represent a heavy burden to patients, their families, and an economic burden on the healthcare system.

**Objective:**

The aim of this study was to examine patterns of trauma-related rehospitalization within 7, 30, 60, and 90 days after initial discharge, and to assess the association between patient age and the risk of rehospitalization, while adjusting for relevant demographic and clinical factors.

**Methods:**

This retrospective cohort study links two national databases: The Israeli National Trauma Registry and the Ministry of Health’s hospitalization database. Univariate analyses (χ^2^ tests) were performed to identify factors associated with rehospitalization at 7, 30, 60, and 90 days post-discharge. Variables significantly associated in univariate analysis were entered into separate multivariable Generalized Estimating Equations (GEE) models for each time point.

**Results:**

The study included 297,022 trauma patients (median age 34 years [IQR: 14–67]; 59.3% male). Most injuries were non-penetrating (88.8%), and the majority were classified as mild (ISS 1–8, 64.9%). Falls (53.0%) and road traffic accidents (23.8%) were the most common injury mechanisms. The most affected age group was 19–54 years (34.3%), and 71.3% of patients were from the Jewish population group.

The rate was 4.4% for rehospitalized within seven days, 9.2% within 30 days, 12.3% within 60 days, and 14.7% within 90 days. In multivariable analysis, a clear association was observed between increasing age and higher odds of rehospitalization following discharge for trauma. Compared to the reference group (ages 0–4), older patients exhibited substantially elevated odds across all follow-up periods. For instance, patients aged 75–84 had odds ratios (ORs) of 4.39, 4.75, 4.95, and 3.33 for rehospitalization within 7, 30, 60, and 90 days, respectively. Similarly, individuals aged 85 + had ORs of 5.42, 5.68, 6.0, and 3.47, respectively. Higher odds of rehospitalization peaked at 60 days post-discharge and decreased by 90 days, suggesting possible stabilization.

**Conclusions and recommendations:**

Comprehensive discharge planning and a care transition system for trauma patients should involve a multidisciplinary team to address the patient's specific condition and rehospitalization risk factors, especially among older adults hospitalized following a fall.

## Introduction

As the global population continues to grow and age, the burden of injury-related hospitalizations on healthcare systems has become increasingly pronounced. This necessitates urgent attention and a comprehensive investigation. Unplanned rehospitalizations place a significant emotional burden on patients and their families, while also imposing substantial economic costs on the healthcare system [1, 2]. In line with existing literature, the terms "readmission" and "rehospitalization" are used interchangeably to refer to any unplanned return to hospital care following discharge.

The 30-day rehospitalization rate, in particular, is widely used as a quality indicator for hospital care systems [2], including trauma care [3]. Other quality indicators, such as rehospitalization rates within 7 or 60 days, are also utilized. Notably, short-term rehospitalizations (within 7 days) and long-term rehospitalizations (within 30 or 60 days) are influenced by different risk factors. For instance, an Australian study of 62,235 medical discharges found that length of stay and advanced age were associated with long-term rehospitalization risk, whereas early rehospitalizations were more influenced by time since last surgery, discharge ward, and discharge timing (day of the week and time of the day of admission and discharge) [1].

Published 30-day rehospitalization rates after trauma range widely, from approximately 6% to 30% [3–11]. For example, an analysis of over 250,000 trauma discharges in California found a 7.6% rehospitalization rate, with more than one-third occurring at a hospital different from the original one [7]. Key significant predictors included discharge against medical advice (odds ratio (OR = 2.56); Charlson score [12] of 2 or higher (OR = 2.00); and age 45 years or older (OR = 1.29). Rehospitalizations were often due to musculoskeletal issues (22.3%), psychiatric conditions (9.4%), and surgical infections (6.7%) [7]. Another Australian study reported a 6.3% rehospitalization rate following lower limb orthopedic trauma in motorcycle accidents, primarily due to fracture care, infections, or post-operative complications [11]. A U.S. study of 2,411 trauma patients at a Level I trauma center reported a 6% 30-day rehospitalization rate, with 57% returning to the index hospital [13]. Among patients with traumatic brain injury (TBI), 35% were readmitted, and 40% of these rehospitalizations occurred within 30 days [14]. The most common diagnoses were subdural hematoma, septicemia, urinary tract infection, and aspiration.

Pediatric trauma patients generally exhibit lower rates of hospital rehospitalization compared to adults in documented literature. In one U.S. cohort, only 1.7% of pediatric trauma patients experienced an unplanned 30-day rehospitalization, with a similar rate (1.8%) among those who underwent surgery during the initial admission [15, 16]. However, higher rehospitalization rates were observed among children with more severe injuries—3.4% in those with Injury Severity Scores (ISS) of 16–24 and 4.9% for ISS ≥ 25—as well as in those with prolonged hospital stays, severe abdominal or pelvic injuries (3.0%), crushing injuries (2.8%), firearm injuries (4.5%), or fluid and electrolyte disorders (3.9%). Among readmitted patients, nearly 39% required surgery, most commonly involving the musculoskeletal system. These findings suggest that although pediatric rehospitalizations are relatively infrequent, they often indicate serious complications and may be concentrated in specific high-risk subgroups [15]. Nevertheless, evidence increasingly highlights that the most vulnerable trauma patients are elderly individuals, particularly those admitted following falls. As this population continues to grow, it is likely to contribute disproportionately to future rehospitalization rates [17]. In one study, their 30-day rehospitalization rate was 17%, and 15% of those readmitted were hospitalized due to another fall [17]. Socioeconomic disadvantages are also a relevant factor: low SES was associated with increased 90-day rehospitalization rates among elderly patients with hip fractures [18].

A significant proportion of rehospitalizations may be preventable [19]. One study showed that a trauma transitional care coordination program halved the hospital's rehospitalization rate compared to the previous year [20]. A Trauma Transitional Care Coordination Program is a healthcare service that helps trauma patients after they leave the hospital. A nurse or coordinator checks in with patients soon after discharge to support their recovery, help with medications and appointments, and prevent problems that could lead to rehospitalization [21, 22]. Furthermore, another study published in 2021 found that 22.7% of trauma patient rehospitalizations were potentially preventable, highlighting the need for targeted interventions to reduce unnecessary hospitalizations and associated healthcare costs. This analysis utilized the 2021 Nationwide Rehospitalizations Database (NRD), the largest publicly available rehospitalizations database in the U.S., which includes data from 30 geographically dispersed states, accounting for 61.2% of the U.S. population and 59.6% of all U.S. hospitalizations [19].

For elderly patients, fall prevention strategies should be implemented after every fall-related admission [23]. Early discharge planning, including functional assessments, patient and caregiver education, medication review, and post-discharge follow-up, has been shown to reduce rehospitalizations by 22% and shorten rehospitalization stays by nearly 2.5 days compared to usual care [24].

While previous studies have examined 30-day rehospitalization rates among trauma patients, most have focused on single institutions, specific injury types, or limited follow-up periods. In addition, few studies have simultaneously assessed multiple rehospitalization timeframes (e.g., 7, 30, 60, and 90 days) within a population-based cohort. Furthermore, limited data are available from non-U.S. settings, particularly in countries with universal healthcare systems. This study addresses these gaps by analyzing national-level data from Israel and exploring short- and longer-term rehospitalization patterns across diverse trauma subgroups. The findings aim to provide a more nuanced understanding of rehospitalization dynamics and inform strategies for post-discharge care and prevention.

## Objective

The aim of this study was to examine patterns of trauma-related rehospitalization within 7, 30, 60, and 90 days after initial discharge, and to assess the association between patient age and the risk of rehospitalization, while adjusting for relevant demographic and clinical factors.

## Method

### Study design

This retrospective cohort study links two national databases: The Israeli National Trauma Registry (INTR) and the Ministry of Health database on hospitalizations.

### Study population

During the study period (between January 1, 2010, and December 31, 2018), 318,667 patients were included in the Israeli National Trauma Registry (INTR), which provides comprehensive data on hospitalized trauma patients from all six Level I Trauma Centers (TC) and 14 (out of 20) Level II TCs in Israel. The INTR comprises all injured individuals who arrived at the hospital within 72 h after the injury event and were either admitted to the hospital or died in the hospital (including deaths occurring in the emergency department). The INTR does not include injuries resulting from poisoning, drowning, suffocation, patients discharged from the hospital after treatment in the emergency room, or injured individuals declared dead before arriving at the emergency room.

**Fig. 1:**
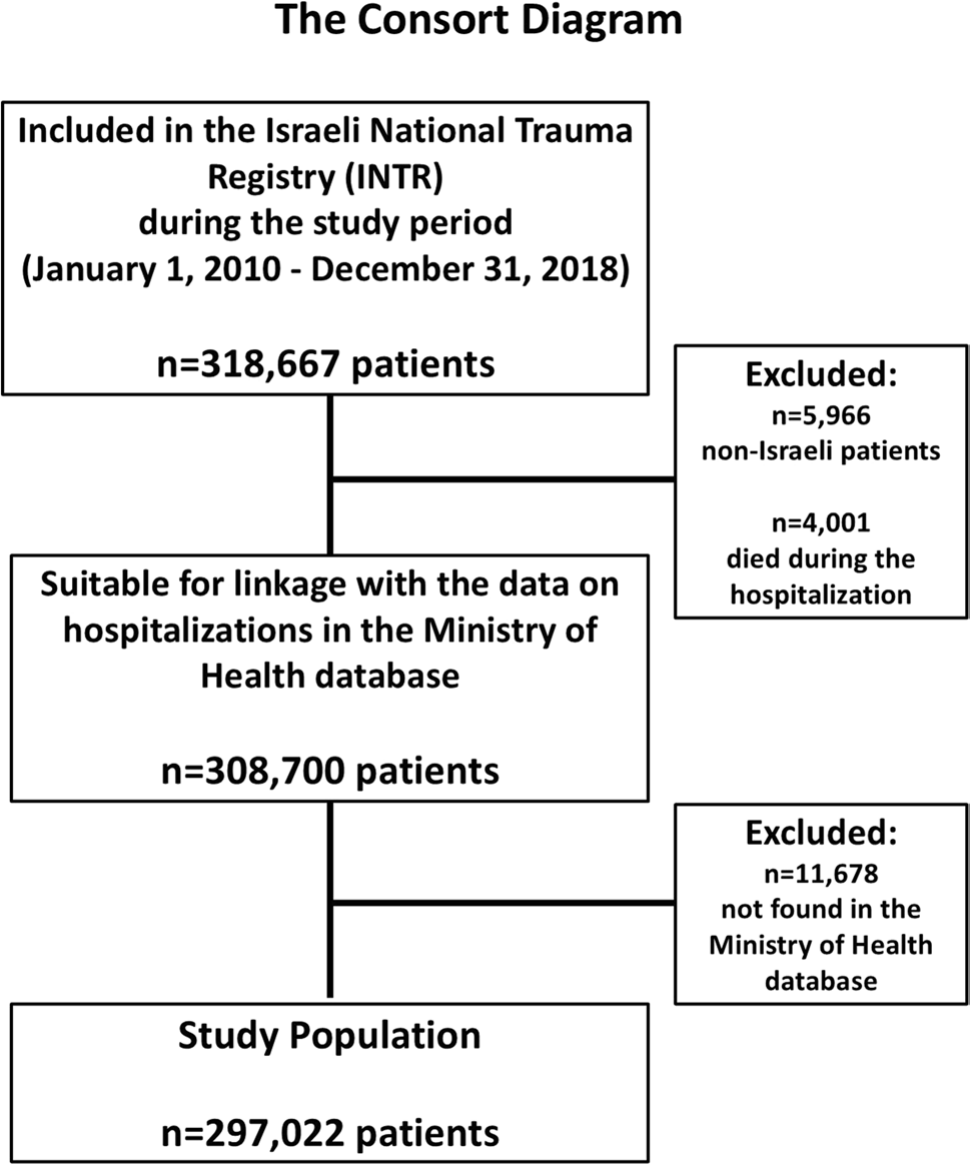
The consort diagram

As non-Israeli citizens do not possess a Teudat Zehut (Israeli identity card), it is not possible to cross-reference their data with other databases; therefore, 5,966 non-Israeli patients were excluded due to linkage limitations. Additionally, 11,678 individuals could not be matched with the Ministry of Health database. Together, 17,644 patients were excluded for these reasons. Patients who died during the initial hospitalization (*n* = 4,001) were also excluded. After these exclusions, data of 297,022 patients from the INTR who were discharged alive after initial hospitalization following traumatic injury were successfully linked with all hospitalizations in the Ministry of Health database (Fig. 1).

The excluded group and the study population were comparable across most variables, with only minor differences (typically within ± 2%) that are unlikely to be clinically meaningful. However, the excluded group had a significantly higher proportion of children aged 0–14 (+ 20.1%) and fewer adults aged 15–64 (–15.7%). This age distribution difference was statistically significant, as determined by a chi-square test (*p* < 0.001).

Data recorded in the INTR includes patient demographic details, injury characteristics (diagnosis and circumstances), hospital resource utilization, length of stay (LOS), and disposition. The Injury Severity Score (ISS) is automatically calculated based on Abbreviated Injury Scale (AIS) codes assigned by trained trauma registrars at each trauma center. Injury data are entered into an electronic system that computes the ISS, and all records undergo national-level validation by the Israel National Center for Trauma and Emergency Medicine Research to ensure accuracy and consistency [25].

### Study variables

Rehospitalization was defined as the hospitalization of the same patient within 90 days from the initial discharge after trauma-related hospitalization. The duration of time out of the hospital was calculated as the difference between the rehospitalization date and the discharge date. Four dichotomous variables (rehospitalization within seven days, 30 days, 60 days, and 90 days were constructed. Only the first rehospitalization following discharge from the initial trauma-related hospitalization was considered in this study. Any subsequent rehospitalizations beyond the first were not included in the analysis. Furthermore, rehospitalization in this study refers to rehospitalization to any hospital, not necessarily the initial (index) hospital.

Demographic variables were collected from the INTR, including age, sex, and population group. The variable age was categorized into eight predefined groups to enable the assessment of rehospitalization trends across life stages. The age groups were defined based on both clinical relevance and public health considerations to reflect meaningful differences in trauma patterns, healthcare needs, and risk of rehospitalization across the lifespan. In addition to the categorical variable, the median age and interquartile range (IQR) were calculated for the overall study population and for each rehospitalization interval (within 7 days, 30 days, 60 days, and 90 days).

The population group variable classified patients by ethno-religious background into the following categories: Jewish and non-Jewish. This variable was included to assess differences in injury characteristics and rehospitalization rates across demographic segments of the population.

The injury mechanism was categorized into five groups: burns, road traffic accidents (RTA), intentional, falls, and other unintentional injuries ("struck by objects or persons","cuts","injury caused by machines", and"other unintentional and unknown causes").

Injury Type was dichotomized as penetrating or non-penetrating.

The Injury Severity Score (ISS) was calculated as the sum of the squares of the single highest Abbreviated Injury Scale score for each of the three most severely injured body regions [26]. Injury severity was then categorized as follows: 1–8 (mild injury), 9–14 (moderate injury), 16–24 (severe injury), and 25 + (critical injury) [27].

The injured body region was represented by six dichotomous variables, indicating the presence or absence of injury in the following regions: head and neck, face, chest, abdomen, extremities, and external injuries. For each body region, we also created dichotomous variables indicating whether the Abbreviated Injury Scale (AIS) score was 3 or higher (yes vs. no).

Traumatic Brain Injury (TBI) was defined based on the AIS score. Severe TBI was defined as AIS ≥ 5, moderate TBI as AIS 3–4, mild TBI as AIS 1–2, and no TBI as AIS 0 [28].

### Statistical analysis

This study used both univariate and multivariable statistical methods to examine the association between the age of the patients and the likelihood of rehospitalization at multiple time points following discharge. Univariate analyses were performed to identify demographic/clinical trends in readmissions within seven days, 30 days, 60 days, and 90 days, using the χ^2^ test. Before including possible confounders in the multivariable analysis, the correlation between the variables was checked with Kendall's Tau coefficient. In the multivariable analysis, 598 patients (0.02%) with missing data were excluded.

To account for clustering of patients within hospitals, we used Generalized Estimating Equations (GEE) with a logit link function to model the binary outcome of rehospitalization within seven days, 30 days, 60 days, and 90 days (yes = 1, no = 0). GEE is a population-averaged modeling approach that adjusts for intra-cluster correlation and was implemented using the geeglm() function from the geepack package in R. Age, sex, population group, injury mechanism, ISS, and the type of injury were included in the multivariable model. The hospital variable was used as the clustering variable to account for correlations among patients treated at the same facility. An exchangeable correlation structure was assumed, reflecting constant correlation among patients within each hospital. Results are reported as odds ratios (ORs) with 95% confidence intervals (CIs), and a *p*-value < 0.05 was considered statistically significant.

All statistical analyses were performed using R software (version 4.1.0), and a *p*-value of two-sided testing less than 0.05 was considered statistically significant.

This study was conducted in accordance with the Declaration of Helsinki and approved by the Ethical Committee of the Sheba Medical Center (#SMC-5424–18) and the Ministry of Health (#20187712), which determined that informed consent was not required due to the use of anonymous data.

## Results

The distribution of demographics and index injury characteristics among 297,022 patients hospitalized in the study period is presented in Table 1.

**Table 1:** The proportion of rehospitalized patients by demographic and injury characteristics

Characteristics of the injured and the injury	Total		Patients readmission											
			Within 7 Days			Within 30 days			Within 60 days			Within 90 days		
	*n*	col %	*n*	%	row %	*n*	col %	row %	*n*	col %	row %	*n*	col %	row %
Total	297,022	100	13,187	100	4.4	27,260	100	9.2	36,600	100	12.3	43,551	100	14.7
Age, years														
0–4	32,365	10.9	464	3.5	1.4	951	3.5	2.9	1,267	3.5	3.9	1,564	3.6	4.8
5–14	43,304	14.6	944	7.2	2.2	1,834	6.7	4.2	2,325	6.4	5.4	2,683	6.2	6.2
15–18	15,900	5.4	573	4.3	3.6	1,008	3.7	6.3	1,251	3.4	7.9	1,448	3.3	9.1
19–54	101,861	34.3	4,350	33.0	4.3	9,690	35.5	9.5	13,535	37.0	13.3	16,626	38.2	16.3
55–64	23,211	7.8	1,066	8.1	4.6	2,158	7.9	9.3	2,802	7.7	12.1	3,251	7.5	14.0
65–74	23,885	8.0	1,433	10.9	6.0	2,805	10.3	11.7	3,659	10.0	15.3	4,265	9.8	17.9
75–84	31,967	10.8	2,232	16.9	7.0	4,623	17.0	14.5	6,171	16.9	19.3	7,204	16.5	22.5
85 +	24,529	8.3	2,125	16.1	8.7	4,191	15.4	17.1	5,590	15.3	22.8	6,510	14.9	26.5
Age, years median (IQR)	34.0 (14.0–67.0)		58.0 (27.0–80.0)			55.0 (28.0–79.0)			54.0 (27.0–79.0)			53.0 (27.0–79.0)		
Sex														
Male	176,102	59.3	7,182	54.5	4.1	13,958	51.2	7.9	17,836	48.7	10.1	20,653	47.4	11.7
Female	120,915	40.7	6,004	45.5	5.0	13,299	48.8	11.0	18,761	51.3	15.5	22,895	52.6	18.9
Population group														
Jewish	211,670	71.3	10,223	77.5	2.8	21,175	77.7	10	28,486	77.8	13.5	33,832	77.7	16
Non-Jewish	84,220	28.4	2,903	22.0	3.4	5,968	21.9	7.1	7,966	21.8	9.5	9,546	21.9	11.3
Missing	1,132	0.4	61	0.5	5.4	117	0.4	10.3	148	0.4	13.1	173	0.4	15.3
Injury mechanism (index admission)														
Burns	8,564	2.9	166	1.3	1.9	287	1.1	3.4	361	1.0	4.2	417	1.0	4.9
Road Traffic Accidents	70,830	23.8	2,669	20.2	3.8	5,808	21.3	8.2	8,102	22.1	11.4	9,917	22.8	14.0
Intentional	14,546	4.9	523	4.0	3.6	976	3.6	6.7	1,262	3.4	8.7	1,511	3.5	10.4
Falls	157,468	53.0	8,443	64	5.4	17,415	63.9	11.1	23,197	63.4	14.7	27,417	63	17.4
Other non-intentional*	45,345	15.3	1,372	40.4	3.0	2,753	10.1	6.1	3,652	10.0	8.1	4,255	9.8	9.4
Missing	249	0.1	13	0.1	5.2	21	0.1	7.6	26	0.1	9.6	34	0.1	12.4
Injury type														
Non-penetrating	263,618	88.8	12,161	92.2	4.6	25,314	92.9	9.6	34,093	93.2	12.9	40,668	93.4	15.4
Penetrating	33,404	11.2	1,026	7.8	3.1	1,946	7.1	5.8	2,507	6.8	7.5	2,883	6.6	8.6
Injury severity score (index admission)														
Mild (ISS 1–8)	192,316	64.9	6,860	52.2	3.6	15,125	55.6	7.9	20,844	57.1	10.8	25,358	58.4	13.2
Moderate (ISS 9–14)	81,633	27.5	4,912	37.4	6.0	9,423	34.7	11.5	12,301	33.7	15.1	14,199	32.7	17.4
Severe (ISS 16–24)	14,934	5.0	875	6.7	5.9	1,668	6.1	11.2	2,094	5.7	14.0	2,409	5.5	16.1
Critical (ISS 25 +)	7,555	2.5	498	3.8	6.6	971	3.6	12.9	1,276	3.5	16.9	1,485	3.4	19.7
Body region injured (index admission)														
Head and Neck	40,063	13.5	1,706	12.9	4.3^(p=.058)^	3,232	11.9	8.1	4,195	11.5	10.5	4,910	11.3	12.3
Face	20,655	7.0	821	6.2	4.0	1,595	5.9	7.7	2,066	5.6	10	2,445	5.6	11.8
Chest	24,427	8.2	958	7.3	3.9	2,008	7.4	8.2	2,584	7.1	7.1	3,023	6.9	12.4
Abdomen	16,905	5.7	702	5.3	4.2^(p=.062)^	1,422	5.2	8.4	1,831	5.0	10.8	2,130	4.9	12.6
Extremities	157,382	53.0	9,024	68.4	5.7	17,571	64.5	11.2	22,437	61.3	14.3	25,878	59.4	16.4
External injury	116,247	39.1	3,572	27.1	3.1	8,656	31.8	7.4	12,828	35.0	11	16,005	36.8	13.8
Body region injured (AIS 3+) (index admission)														
Head and Neck	22,616	7.6	1,256	9.5	5.6	2,358	8.7	10.4	3,031	8.3	13.4	3,500	8.0	15.5
Face	678	0.2	27	0.2	4.0^(p=.563)^	61	0.2	9.0^(p=.870)^	86	0.2	12.7^(p=.774)^	98	0.2	14.5^(p=.878)^
Chest	14,997	5.0	654	5.0	4.4^(p=.630)^	1,352	5.0	9.0^(p=.479)^	1,710	4.7	11.4	1,994	4.6	13.3
Abdomen	5,602	1.9	322	2.4	5.7	641	2.4	11.4	811	2.2	14.5	933	2.1	16.7
Extremities	65,774	22.1	4,343	32.9	6.6	8,369	30.7	12.7	11,035	30.2	16.8	12,774	29.3	19.4
External injury	1,353	0.5	33	0.3	2.4	72	0.3	5.3	97	0.3	7.2	114	0.3	8.4
Traumatic brain injury (index admission)														
No brain injury	228,033	76.8	10,818	82	4.7	22,506	82.6	9.9	30,217	82.6	13.3	35,926	82.5	15.8
Mild brain injury	47,428	16.0	1,164	8.8	2.5	2,499	9.2	5.3	3,489	9.5	7.4	4,287	9.8	9.0
Moderate brain injury	18,414	6.2	933	7.1	5.1	1,781	6.5	9.7	2,269	6.2	12.3	2,602	6.0	14.1
Severe brain injury	3,147	1.1	272	2.1	8.6	474	1.7	15.1	625	1.7	19.9	736	1.7	23.4

* Other unintentional injuries include injuries from objects and people that occurred without any intention of causing damage to oneself or others

Unless otherwise specified, all *p*-values in the table are <0.01. Exact *p*-values are provided for non-significant results

The median age was 34 years (IQR: 14–67). The majority of the patients were male (59.3%) and Jewish (71.3%).

Regarding injury mechanisms, falls were the most common cause of injury (53%), followed by road traffic accidents (23.8%). The most frequently injured body region was the extremities (53%), while external injuries were present in 39.1% of the cases.

Most patients experienced non-penetrating trauma (88.8%), and the injuries were predominantly classified as mild (ISS 1–8, 64.9%). A smaller proportion sustained moderate (27.5%), severe (5.0%), or critical injuries (2.5%). Concerning traumatic brain injury (TBI), 76.8% had no TBI, while 16.0% had mild, 6.2% moderate, and 1.1% severe TBI.

**Fig. 2:**
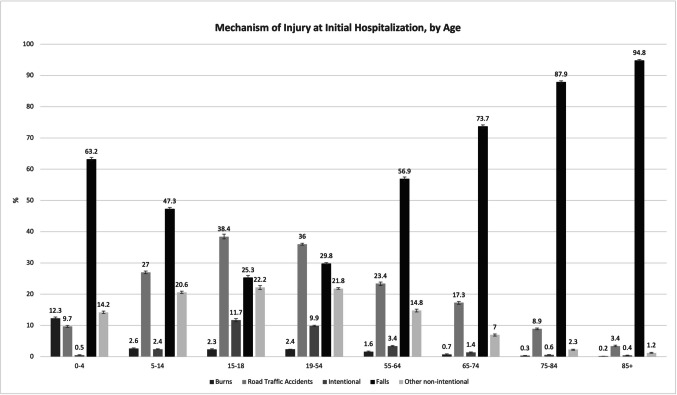
Mechanism of injury at initial hospitalization, by age. Note: Error bars represent 95% confidence intervals

Figure 2 illustrates a clear age-related trend in the mechanism of injury at the time of initial hospitalization. Falls were the most frequent mechanism in all age groups and became increasingly dominant with age. In the youngest group (0–4 years), falls accounted for 63.2% of injuries, while in the oldest group (85 +), this proportion rose sharply to 94.8%. This trend begins to solidify from the age of 55 onward, with falls comprising 56.9% of injuries among individuals aged 55–64, 73.7% among those aged 65–74, and reaching 87.9% in those aged 75–84. In contrast, road traffic accidents (RTAs) and intentional injuries were more prominent among adolescents and young adults. For example, RTAs accounted for 38.4% of injuries in the 15–18 age group and 36% in the 19–54 age group. Intentional injuries peaked at 25.3% in adolescents (15–18), then declined significantly in older age groups.

Burns were most common among children aged 0–4 (12.3%) and decreased dramatically with age, falling below 1% in those aged 65 and older.

### Univariate analysis of rehospitalization due to any cause

A total of 43,551 patients (14.7%) were rehospitalized within 90 days following their initial injury. Rehospitalization rates increased over time: 4.4% within 7 days, 9.2% within 30 days, and 12.3% within 60 days. (Table 1). The median age of rehospitalized patients was significantly higher than that of the total cohort (median: 53–58 years vs. 34 years in the full cohort).

Rehospitalization rates rose with age, reaching 26.5% among those aged 85 years and older, compared to only 4.8% among children aged 0–4 years. Notably, females had a higher 90-day rehospitalization rate (18.9%) than males (11.7%).

Among population groups, non-Jewish patients had a higher rate of 90-day rehospitalization (11.3%) compared to Jewish patients (10%). Falls, as already established as the most common injury among older adults, were also strongly associated with higher rehospitalization rates. Of the total population, patients with fall-related injuries had the highest proportion of readmissions: 17.4% within 90 days, compared to 14.0% for RTAs and only 4.9% for burns.

Patients with penetrating injuries had consistently lower rehospitalization rates than those with non-penetrating injuries.

As expected, rehospitalization increased with injury severity: 90-day rates were 13.2% for mild injuries, 17.4% for moderate, 19.7% for critical injuries, and 23.4% for severe traumatic brain injury.

In terms of anatomical injury location, injuries to the extremities were most strongly associated with repeated hospitalization, with a 90-day rehospitalization rate of 16.4%, followed by injuries to the abdomen (12.6%) and head and neck (12.3%). Similarly, patients with AIS 3 + extremity injuries had a notably high rehospitalization rate of 19.4%.

### Multivariable analysis of rehospitalization due to any cause

After adjusting for within-hospital clustering using Generalized Estimating Equations (GEE), a statistically significant association was observed between age and the likelihood of rehospitalization at all time points (Table 2).

**Table 2:** Generalized estimating equations (GEE) model for rehospitalization, by patient and injury characteristics

Characteristics of the injured and the injury	Odds ratio (OR) and 95% confidence interval (CI) for rehospitalization since discharge within (# of days):			
	7 days	30 days	60 days	90 days
Demographic and other characteristics				
Age				
0–4	1.0*	1.0*	1.0*	1.0*
5–14	**1.59** **1.41–1.78**	**1.53** **1.41–1.66**	**1.48** **1.37–1.59**	**1.73** **1.56–1.92**
15–18	**2.81** **2.47–3.18**	**2.56** **2.33–2.81**	**2.46** **2.26–2.67**	**3.09** **2.75–3.46**
19–54	**3.31** **3.00–3.66**	**3.84** **3.58–4.13**	**4.21** **3.95–4.48**	**3.32** **3.03–3.65**
55–64	**3.19** **2.85–3.57**	**3.30** **3.05–3.58**	**3.29** **3.07–3.53**	**3.42** **3.08–3.79**
65–74	**3.96** **3.55–4.41**	**3.96** **3.67–4.28**	**3.97** **3.71–4.26**	**3.25** **2.93–3.61**
75–84	**4.39** **3.96–4.88**	**4.75** **4.41–5.11**	**4.95** **4.64–5.29**	**3.33** **3.01–3.68**
85 +	**5.42** **4.88–6.03**	**5.68** **5.26–6.12**	**6.00** **5.61–6.41**	**3.47** **3.13–3.86**
Sex (Female vs. Male)	**0.84** **0.81–0.87**	**1.27** **1.24–1.30**	**1.11** **1.08–1.14**	**0.95** **0.91–0.98**
Population group (Jews vs. non-Jews)	**1.04** **1.00–1.09**	**1.05** **1.02–1.09**	**1.05** **1.02–1.08**	**1.05** **1.00–1.09**
Injury and hospitalization characteristics				
Injury mechanism (index admission)				
Burns	1.0*	1.0*	1.0*	1.0*
RTA	**1.23** **1.05–1.45**	**1.64** **1.45–1.86**	**1.87** **1.67–2.10**	**1.22** **1.06–1.42**
Intentional	1.18 0.99–1.42	**1.41** **1.22–1.62**	**1.53** **1.35–1.74**	1.07 0.90–1.26
Falls	**1.59** **1.36–1.87**	**2.10** **1.86–2.38**	**2.31** **2.07–2.59**	**1.67** **1.45–1.93**
Other non-intentional	**1.23** **1.04–1.45**	**1.53** **1.35–1.75**	**1.70** **1.51–1.91**	**1.31** **1.12–1.53**
Unknown	**1.92** **1.06–3.46**	**1.75** **1.07–2.87**	**1.86** **1.19–2.89**	1.33 0.71–2.49
ISS				
Mild Injury (ISS 1–8)	1.0*	1.0*	1.0*	1.0*
Moderate Injury (ISS 9–14)	**1.21** **1.16–1.26**	**1.04** **1.01–1.07**	0.97 0.95–1.00	**0.91** **0.87–0.95**
Severe Injury (ISS 16–24)	**1.37** **1.27–1.47**	**1.19** **1.13–1.26**	**1.09** **1.04–1.14**	**1.21** **1.12–1.30**
Critical Injury (ISS 25 +)	**1.66** **1.51–1.83**	**1.50** **1.40–1.62**	**1.46** **1.37–1.56**	**1.66** **1.52–1.82**
Injury type				
Non-penetrating	1.0*	1.0*	1.0*	1.0*
Penetrating	**0.87** **0.81–0.95**	**0.79** **0.75–0.84**	**0.75** **0.71—0.79**	**0.91** **0.84–0.98**

^*^ Reference group (OR = 1.0)

^**^Statistically significant values are shown in **bold**

In multivariable analysis, a clear association was observed between increasing age and higher odds of rehospitalization following discharge for trauma. Compared to the reference group (ages 0–4), older patients exhibited substantially elevated odds across all follow-up periods. For instance, patients aged 75–84 had odds ratios (ORs) of 4.39, 4.75, 4.95, and 3.33 for rehospitalization within 7, 30, 60, and 90 days, respectively. Similarly, individuals aged 85 + had ORs of 5.42, 5.68, 6.0, and 3.47, respectively. Higher odds of rehospitalization peaked at 60 days post-discharge and decreased by 90 days, suggesting possible stabilization.

### Rehospitalization following injury

Among those who were readmitted within 90 days (*n* = 43,551), 31% (*n* = 13,523) were hospitalized following another traumatic injury. Among them, the mean length of stay during the second hospitalization was 6.3 days (SD = 12.6); median 3 days [IQR 1–6]. The death rate during the hospitalization was 0.1% (*n* = 16).

**Fig. 3:**
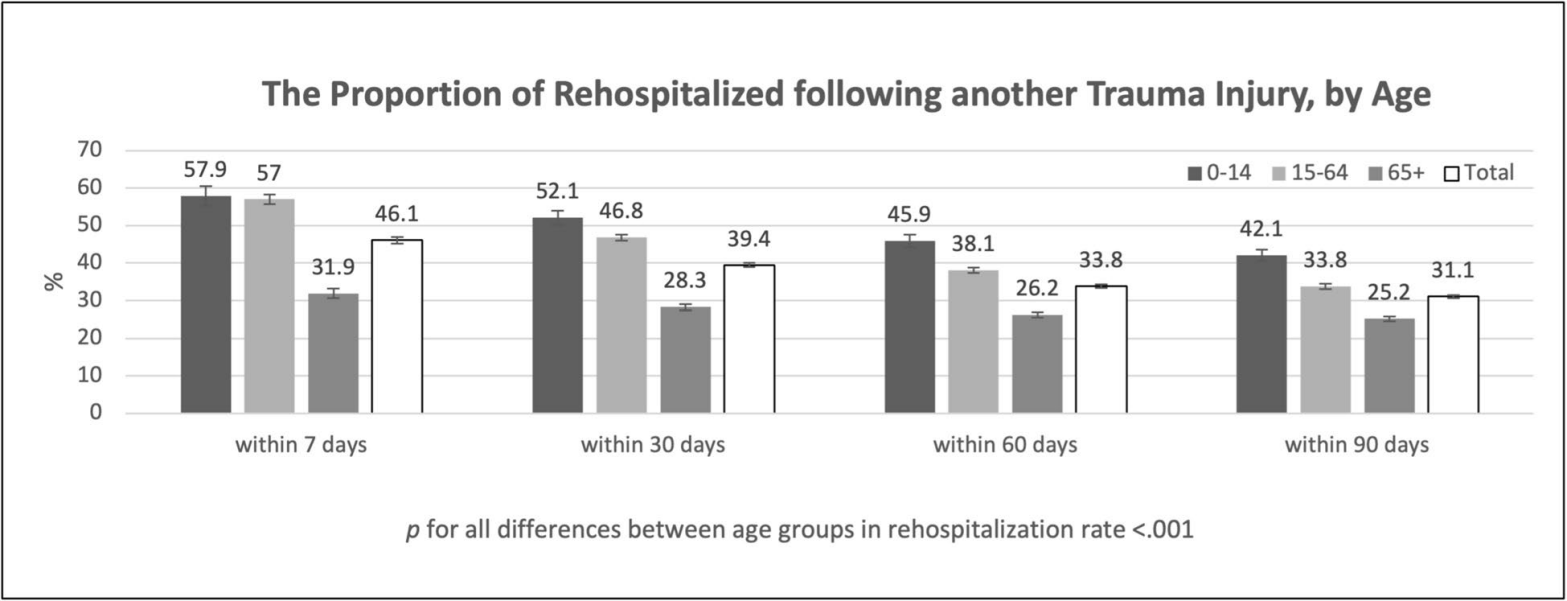
The proportion of rehospitalized following another trauma injury, by age. Note: Error bars represent 95% confidence intervals

Specifically, 42.1% of those aged 0–14 years were hospitalized following a traumatic injury; among those aged 15–64 years, the proportion was 33.8%; and among those aged 65 and older, the proportion of rehospitalizations following trauma was 25.2% (Fig. 3).

**Fig. 4:**
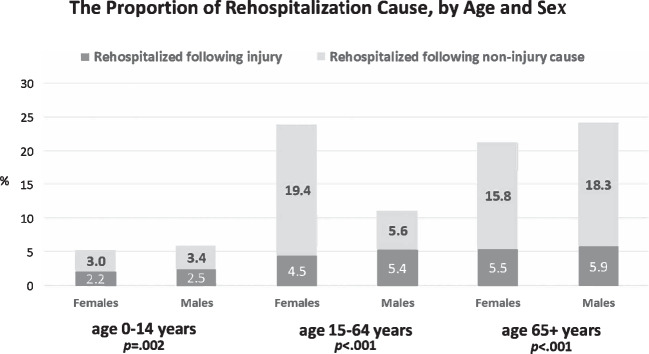
The proportion of rehospitalization cause, by age and sex

Regarding the risk of rehospitalization following injury, males of all age groups had a significantly higher risk compared to females (2.5% vs. 2.2% among those aged 0–14; 5.4% vs. 4.5% among those aged 15–64 and 5.9% vs. 5.5% among those aged 65 and older) (Fig. 4). The risk of rehospitalization following injury was higher among males (as shown in Fig. 4).

## Discussion

The study aimed to identify the risk factors for rehospitalization among trauma patients within 7 days, 30 days, 60 days, and 90 days after discharge. The rehospitalization rate within 90 days in this study (14.7%) was within the range reported in previous studies on trauma patients (6% to 30%) [3–11]. Compared to the youngest group, older patients exhibited substantially elevated odds across all follow-up periods. These findings are consistent with previous studies demonstrating a similar relationship between age and rehospitalization rates. For instance, Moore et al. found that older trauma patients had significantly higher rehospitalization rates within any period post-discharge compared to younger patients [6]. Similarly, Li and colleagues reported that age was a strong predictor of rehospitalization in a large cohort of trauma patients, with older individuals exhibiting double the risk compared to younger cohorts [29]. These studies, along with our findings, highlight the persistent and significant impact of age on post-trauma recovery outcomes. The increased odds of rehospitalization with advancing age can be attributed to several factors. Older adults are more likely to have pre-existing comorbidities, such as cardiovascular diseases, diabetes, and chronic respiratory conditions, which can complicate recovery from trauma and increase the need for subsequent medical interventions [6]. Furthermore, age-related physiological changes, such as reduced immune function and slower tissue repair, can impede the healing process and contribute to higher rehospitalization rates [30]. Additionally, older individuals may face challenges such as decreased mobility, reduced physical resilience, and higher susceptibility to infections, further complicating their recovery. The presence of cognitive impairments and decreased functional capacity can also impact their ability to adhere to post-discharge care plans, leading to higher rates of complications and rehospitalizations [31, 32].

Most older adults are hospitalized following a fall, so our findings underscore the importance of age-specific post-trauma care and rehabilitation strategies. Healthcare providers should consider implementing tailored interventions for older patients to address their unique medical needs and mitigate the risk of rehospitalization. Strategies may include comprehensive discharge planning, closer follow-up care, enhanced rehabilitation programs, and additional support services to ensure adherence to treatment and recovery plans. Additionally, it is essential to prepare the environment for discharged patients. We found that among patients aged 65 years and older, more than a third of those rehospitalized within seven days were readmitted following another trauma. Preventing falls in older adults involves a combination of environmental modifications, lifestyle changes, and medical management [33]. A study conducted in the United States in 2018 included almost 800,000 patients aged 65 and older injured due to falls. Another fall was the third leading cause of rehospitalization after septicemia and heart failure [34], which is similar to our current findings. The authors concluded that intervention strategies focused on recurrent fall prevention during and after hospitalization may reduce the risk of rehospitalization. Of course, not all rehospitalizations are preventable. A study that determined the preventability of rehospitalization among hospitalized patients aged 55 and older (not only trauma patients) found that approximately one-quarter of rehospitalizations are potentially preventable by greater attention to patients'readiness for discharge, enhanced disease monitoring, and better support for patient self-management [35]. Still, as falls are an essential cause of rehospitalization, hospital workers should better prepare patients for discharge and maintain contact with the family physician for further ongoing follow-up. Timely referrals for vision-related problems such as cataract operations, participation in balance and strengthening exercises, and osteoporosis treatment can reduce the risk of falls [36]. Additionally, easy and inexpensive improvements in the living space, such as home safety assessment and improvements and modification programs, can significantly reduce fall risk [37]. Transitional prevention strategies to avoid post-discharge falls among patients aged 65 and older are effective [34] and should be implemented in Israel.

Our study revealed the overall gradual increase in the odds of rehospitalization from 7 to 60 days post-discharge, followed by a slight decline at 90 days. The explanation probably lies in the evolving clinical trajectory of trauma recovery. In the first week after discharge, rehospitalizations are likely driven by acute complications directly related to the initial injury or hospital care, such as surgical site infections, bleeding, or early functional deterioration [6]. As time progresses, additional factors emerge—such as delayed complications, unrecognized secondary injuries, psychological stress responses, and challenges with rehabilitation or adherence to discharge instructions—which may account for the elevated odds of rehospitalization observed at 30 and 60 days. However, by 90 days post-discharge, patients who were at the highest risk of complications have already been rehospitalized, and those who remain are more likely to represent a clinically stable subgroup [38].

Regarding younger patients, 8.6% of children aged under 18 years were rehospitalized within one year after trauma in a large cohort of trauma patients [16]. In our study, 6.2% of children in the same age group were rehospitalized within 90 days, which is comparable, considering the length of the follow-up time. In children, trauma recidivism (rehospitalization to hospitalization following another injury) was found to be primarily related to falls (63%) in the previous study [39]. In our study, most of those rehospitalized within seven days and within the first month after discharge were readmitted following an additional trauma. A meta-analysis that included RCTs assessed the influence of parenting intervention programs, possession and use of safety equipment, and safety practices by parents and found these measures effective for preventing injury recidivism in the pediatric population [40].

Another key finding of this study is that there are higher odds of non-injury-related rehospitalization among females, consistent with previous studies [41, 42]. We believe that the finding might be related to biological factors, such as a higher risk of osteoporosis and lower bone density, which increases the risk of fractures and subsequent injuries among females [43], as well as hospitalizations following obstetric reasons. For injury-related rehospitalization, the risk for males was higher than for females, similar to the findings of other studies [44–46]. The higher risk of rehospitalization following injury among males can be attributed to behavioral, biological, and social factors. Males are statistically more likely to engage in high-risk activities, such as extreme sports, heavy manual labor, and hazardous occupations, leading to a higher risk of injury recidivism [47].

To thwart preventable rehospitalizations, a comprehensive discharge planning for trauma patients should involve a team of healthcare professionals, including doctors, nurses, social workers, and pharmacists, to create a discharge plan that addresses the patient's specific condition, recovery goals, and potential risk factors for rehospitalization. Patient discharge should be accompanied by a care transition system with clear communication channels between hospital staff, primary care providers, and community healthcare services to share critical patient information and updates [48]. Health Maintenance Organizations (HMOs) should maintain and conduct post-discharge follow-ups, including follow-up calls or home visits to check on the patient's condition [49]. Furthermore, integrating and using wearable devices or home monitoring systems can be beneficial as follow-up tools [50, 51]. Local authorities should ensure public spaces, such as parks and sidewalks, are well-maintained, well-lit, and free of obstacles that could cause falls [52]. Home safety assessments by trained professionals should be implemented among the high-risk groups (mainly older adults) to identify and mitigate fall hazards, such as poor lighting, slippery floors, and cluttered walkways. Safety and fall prevention workshops are recommended for different age groups, from children to older adults [53]. HMOs may organize these workshops and include exercise programs focusing on improving balance, strength, and flexibility for older adults. Future studies are needed to examine which interventions and practices are most effective in minimizing the risk of rehospitalization.

Several limitations should be considered when interpreting the findings of this study.

First, although we used national-level data from two validated and comprehensive administrative databases, approximately 3.8% of trauma patients listed in the Israeli National Trauma Registry (INTR) could not be linked to the Ministry of Health hospitalization database. These unmatched cases were excluded from the final analysis, potentially introducing a slight selection bias. A comparison between linked and unlinked patients revealed statistically significant differences in demographic and injury characteristics, including age distribution, population group, injury mechanism, and injury severity. These differences suggest potential for selection bias, as the unlinked patients may differ in their risk of rehospitalization. However, the unlinked group comprised only 3.8% of the total cohort; thus, while a minor risk of bias cannot be entirely excluded, its impact on the overall findings is likely limited.

Second, the INTR includes only trauma patients who were hospitalized or died in the hospital (including in the emergency department). As a result, patients treated and discharged directly from the emergency department and individuals who died at the scene or en-route to the hospital are not captured in the dataset. Consequently, our findings may not reflect the full spectrum of trauma severity, particularly in cases of minor injury or extremely severe trauma that never led to hospital admission.

Third, the INTR utilizes ICD-9 external cause of injury codes (E-codes), which characterize the mechanism and intent of injuries. However, the Ministry of Health hospitalization database does not include these codes. Therefore, while we were able to broadly categorize rehospitalization causes as injury-related or non-injury-related, we could not determine the specific causes of rehospitalization.

Fourth, due to the structure of E-coding, injury mechanism and intentionality are combined within a single code, preventing separate analysis of these two variables. Fifth, we were unable to adjust for patients’ underlying comorbidity burden, as the dataset lacked detailed diagnostic information required to compute a comorbidity index such as the Charlson Comorbidity Index. This may have influenced observed associations. Sixth, we were unable to distinguish between elective and unplanned rehospitalizations due to limitations in the available data, nor could we determine whether rehospitalizations were directly related to the initial trauma event.

Finally, the dichotomization of population groups into “Jewish” versus “non-Jewish,” due to limitations in the original data, risks conflating heterogeneous ethno-religious and socioeconomic sub-populations (e.g., Arab Muslim, Christian, Druze). This approach may obscure important within-group disparities and structural inequities. Future research should examine the robustness of these findings using more granular classifications for age and population groups and evaluate their generalizability across diverse populations, healthcare settings, and trauma system structures.

## Conclusions

Despite these limitations, the study revealed that following the initial discharge after injury-related hospitalization, 4.4% of patients were rehospitalized within seven days, 9.2% within 30 days, 12.3% within 60 days, and 14.7% within 90 days. A third of the rehospitalizations occurred following another traumatic injury. Higher odds for rehospitalization were observed among individuals hospitalized due to falls and among older adults. Advanced age and higher injury severity were independently associated with increased rehospitalization risk. These findings underscore the need for targeted post-discharge planning and follow-up strategies, particularly for older adults and those with severe injuries, to reduce preventable rehospitalizations and improve long-term outcomes.

## Data Availability

The data is not available following the decision of the Ethics Committee.
